# Innovations with tele-ultrasound in education sonography: the use of tele-ultrasound to train novice scanners

**DOI:** 10.1186/s13089-021-00210-0

**Published:** 2021-02-14

**Authors:** Anne E. Drake, Jonathan Hy, Gordon A. MacDougall, Brendan Holmes, Lauren Icken, Jon W. Schrock, Robert A. Jones

**Affiliations:** 1 Case Western Reserve University School of Medicine, Health Education Campus, 9501 Euclid Ave, Cleveland, OH 44106 USA; 2 MetroHealth Medical Center, Cleveland, OH USA

**Keywords:** Ultrasound, Medical education, Remote learning, Tele-ultrasound

## Abstract

**Objectives:**

Point-of-care ultrasound (POCUS) has become increasingly integrated into medical education given the growing role of evaluative and procedural techniques in practice today. Tele-ultrasound is a new and promising venture that aims to expand medical knowledge and education to previously unreached or underserved areas. This study aimed to determine the non-inferiority of teaching ultrasound remotely using tele-ultrasound via the Philips Lumify (Philips Medical Systems, Bothell, WA) system, which utilizes video conferencing technology and real-time imaging that can be viewed by the operator and educator simultaneously.

**Methods:**

Three commonly used ultrasound exams were taught and evaluated in 56 ultrasound-naive medical participants: Focused Assessment with Sonography in Trauma (FAST), Lower Extremity Deep Venous Thrombosis (LEDVT) screening, and ultrasound-guided vascular access. The participants were randomized into either in-person traditional learning or tele-ultrasound learning with the Philips Lumify (Philips Medical Systems, Bothell, WA) units. The primary outcome of interest was the ability to perform certain tasks for each exam

**Results:**

Competency on each exam was tested across all exams and no inferiority was found between in-person and remote learning (*p* < 0.05).

**Conclusions:**

Our findings support the use of tele-ultrasound in beginner ultrasound education.

## Introduction

Point-of-care ultrasound (POCUS) has become increasingly integrated into medical education given the growing role of evaluative and procedural techniques in practice today [1]. Previous studies have assessed the value of ultrasound training in medical school education and found sonography training to be engaging and useful to students in their preclinical education and beyond [1–5]. Likewise, access to portable and inexpensive ultrasound devices has continued to increase. These trends in ultrasound access and portability have made training students in bedside ultrasound techniques such as trauma examinations a more readily available component of medical education [4, 6].

To explore the role of tele-ultrasound in medical education, we utilized Philips Lumify (Philips Medical Systems, Bothell, WA) with Reacts Technology (Innovative Imaging Technologies, Montreal, Canada). The Philips Lumify portable ultrasound systems utilize Android tablets, so educators and learners can interact over an internet connection using the Reacts collaborative platform using the front and rear-facing cameras on their devices. The learner can share via live-feed with the educator both the Lumify ultrasound stream as well as the position of the transducer on the patient or model. This allows the educator to help the learner with sonographic anatomy, sonographic findings, and transducer positioning.

The use of tele-ultrasound in education has several interesting applications that make this technology worth investigating. As access to technology grows, there is a relative disparity in the number of educators equipped to teach sonographic techniques, especially in resource-poor areas. This technology could extend the reach of educators further than traditional in-person training. In addition, with the current COVID-19 pandemic ongoing, there has been a push of medical education to adopt a virtual format. Medical schools across the country are searching for ways to adapt an in-person skill to a virtual format. This technology would provide an avenue for students to gain the sonographic skills while maintaining social distancing from their instructor, a process difficult with traditional learning methods.

We aimed to evaluate the non-inferiority of tele-ultrasound to in-person training in the education of medical students who have had no prior ultrasound experience. Students were taught three common ultrasound examinations: the Focused Assessment with Sonography for Trauma (FAST) exam, Lower Extremity Deep Vein Thrombosis (LEDVT) screening, and ultrasound-guided vascular access, with pre- and post-assessments [7].

## Materials and methods

### Participants

Participants included 56 ultrasound-naive first, second, or third-year medical students who had not yet started their clinical rotations. Fourth-year students were excluded because of the possibility of encountering ultrasound within their clinical rotations. Students from three medical schools in the area were recruited via email. Participants were not required to participate and were not compensated for their time. Participants who had experience with the tested exams were excluded, as assessed by a pre-assessment questionnaire. Significant ultrasound experience was also a disqualifying factor, which was defined as over 20 h of lifetime ultrasound experience. The participants were subsequently randomized into traditional in-person (“Traditional”) or tele-ultrasound groups (“Tele-ultrasound”) based on the order of arrival to the simulation center.

### Lumify Ultrasound system

The Lumify Ultrasound system is a portable application-based system that incorporates an ultrasound probe, specially designed to directly connect to a tablet or smart-phone. The S4-1 broadband sector array transducer was utilized for the FAST exam (bandwidth of 4–1 MHz, a scan depth of up to 24 cm, a footprint of 20.2 mm, and imaging features including 2D, color Doppler, and M-mode). For the LEDVT screen and ultrasound-guided vascular access, the L12-4 Linear array transducer (Bandwidth 12–4 MHz, a scan depth of up to 12 cm, a footprint of 34 mm, and imaging features including 2D, steerable color Doppler, M-mode) was utilized. Using the Philips Lumify app, the system allows for “video calls” to another smart device over wireless internet signal. This “call” allows for face-to-face interaction between the operator and educator, allowing the remote instructor to see the positioning of the transducer as well as the generated ultrasound images in real-time. The software additionally includes a pointer that can be utilized by the remote instructor to highlight relevant structures on the ultrasound image.

### Examination techniques

The FAST exam is a bedside examination technique predominantly used in trauma settings to detect the presence of pathologic free fluid, notably allowing for rapid detection of hemoperitoneum in order to initiate and coordinate appropriate interventions. The exam comprised four windows: left upper quadrant, right upper quadrant, pelvic and pericardial windows [8].

The lower extremity deep vein thrombosis (LEDVT) evaluation is important diagnostic tool used for assessment of thrombosis of the common femoral, femoral and popliteal veins. Participants were taught the proximal lower extremity compression technique which begins at the common femoral vein and ends at the trifurcation of the popliteal vein. The comprehensive exam, encompassing continuous compressions every 2.5 cm, was utilized as it is a widely accepted methodology. This method has been previously demonstrated to be a safe, reliable and sensitive method of evaluating for LEDVT [9–12].

Ultrasound-guided vascular access facilitates visualization of the both vessel and needle which is particularly helpful for patients with difficult vasculature with conventional techniques. Ultrasound-guided vascular access was taught in both transverse and longitudinal views, both of which are commonly utilized in clinical practice [13]. A branched 4-vessel ultrasound training phantom (CAE Blue Phantom, Sarasota, FL) was utilized for this exercise.

### Study design

This study was approved by the MetroHealth Medical Center’s Internal Review Board and allowed for verbal consent for participants. Participants who volunteered and met inclusion criteria were then given access to assigned online modules that educated them on basic ultrasound physics and instrumentation as well as each exam that was to be taught. These modules were created by the head of Ultrasound Education at Case Western Reserve University School of Medicine, Dr. Robert Jones. They were presented on emsono.com, an on-demand ultrasound learning and education website that is utilized by many EM residencies to teach ultrasound principles and methods. Modules walked students through important components of the exams with accompanying videos and questions to prepare the students in advance for the study. There was no difference between the modules assigned to the Traditional and Tele-ultrasound groups. Modules included practical scanning, the EFAST exam-hemoperitoneum, vascular access, and lower extremity DVT ultrasound. The total time to complete these modules was approximately 3 h.

Once completed, the participants were instructed to convene on certain dates at pre-specified locations (ex. simulation center at a nearby hospital) and were then randomly assigned to either Traditional or Tele-ultrasound Groups in an alternating fashion based on the order in which they arrived. Both groups received a pre-assessment questionnaire to fill out. This questionnaire was used to assess prior ultrasound experience and expectations of the study. A full breakdown of the questionnaire can be found in Additional file 1: Appendix S1.

The participants were taught the three exams as indicated above. Both groups utilized a 4 participants:1 teacher ratio. The teaching portion of the exam lasted approximately 2 h, with time being divided among the three skills. During this time, all participants were able to practice with the probe and be walked through each exam by the instructor. Students in both groups were given feedback on their hand and probe placement throughout the training period, as well as feedback on what structures needed to be visualized on exam. As these students were considered ultrasound naïve, importance was placed on visualizing and recognizing the structure, with less emphasis on the image quality and ability to operate the ultrasound machine. For a detailed guide on what structures and procedures were considered pertinent for each exam, refer to Additional file 1: Appendix S2.

In the Tele-ultrasound group, students were isolated from the instructor, who was in a separate room within the simulation center. The instructor was kept consistent for all tele-ultrasound participants, and was experienced with the Lumify system. In the Traditional group, students were paired with an experienced sonographer or physician, of which two were utilized for different groups depending on their respective availability. One of three student investigators was utilized as a standardized patient for each exam, with attempts to keep the student-models consistent between the training and exam sessions. The primary outcome of interest at was the ability to perform certain tasks for each exam, a complete breakdown of which can be found in Additional file 1: Appendix S2.

After the teaching portion of the session was complete, the assessment portion began. During this portion, participants rotated between three stations in which they were assessed on the skills they had previously learned. Participants were graded on a pass/fail basis on whether they were able to perform the specified skill. No teaching was done with the participants during the assessment period, and no assistance was given. Medical student investigators were trained by POCUS faculty on the tested exam prior to the session to ensure the student investigators were able to properly assess the participants. The medical student investigators then assessed the participants based on a specified checklist, with the investigators remaining consistent at each station between participants and sessions (i.e., one student investigator assessed all DVT exams performed by participants). This was done to ensure that standards were maintained. The assessment checklists (which can be found in Additional file 1: Appendix S2) mainly consisted of probe choice and important anatomical structures, which had to be visualized sufficiently in order to be considered a “pass”. As specified earlier, since participants with little to no ultrasound experience were recruited, the main goal of this study was to assess whether structures could be visualized and recognized. Participants were not assessed on their ability to calibrate the machine or optimize the image.

### Statistical analysis

The purpose of this study was to show that the tele-ultrasound teaching method was as efficacious as traditional teaching, rather than proving it better or worse. For this reason, a non-inferiority study design was utilized. Outcomes were considered on a “meets” or “does not meet” basis on whether the student was able to perform each designated task for the exam as determined by the medical student investigators who were present. Using the cumulative binary data, means were calculated for each task of the exam. Using the binomial data, a Fisher’s exact test was performed to determine whether inferiority was present. Data were analyzed using STATA v 14.0 (College Station, TX).

Demographic data were collected to ensure adequate randomization between groups. Pre- and Post-assessment surveys were utilized in order to gauge the experience of participants, as well as their subjective opinion on the different systems. Within the pre-assessment, first overall ultrasound experience was assessed, as this was an important excluding factor. Demographic data were collected, and then each participant answered 3 questions relating to their confidence in ability to perform each of the three exams prior to the teaching component of the study. For the post-assessment, both groups had six questions that inquired as to how they felt about their learning experience. The Tele-ultrasound group had two additional questions specifically on the Lumify units and their opinion of remote learning. These questionnaires can be found in Additional file 1: Appendix S3 (Tele-ultrasound) and 4 (Traditional). All surveys utilized the Likert scale, with rankings from 1 (strongly disagree) to 5 (strongly agree). Significant difference between Traditional and Tele-ultrasound groups was calculated using a Mann–Whitney U test.

## Results

Fifty-six participants were recruited for the study, divided equally between the Traditional learning group and Tele-ultrasound Group. Ultimately, all participants successfully completed the study and are included in the data sets. Participants underwent a teaching period first, where they had either in-person or tele-ultrasound training, followed by an assessment period during which each of the three skills was assessed. The skills were assessed by medical student investigators familiar with the skills, and a checklist of important aspects of each exam. The aspects considered important in each exam can be found in Additional file 1: Appendix S2 (Table 1). Demographics of the Tele-ultrasound and Traditional groups were analyzed using the Fisher exact test (significance denoted by *p*-value < 0.05). Significant difference was seen in the number of MS2 students per group, with more MS2 participants in the Traditional group.

**Table 1:** Demographic data on the participants based on group assignment

Variable	Study group		Total	*p*-value
	Traditional (n = 28)	Remote (n = 28)		
Sex				
Male	18 (64.2)	15 (53.6)	33	0.59
Female	10 (35.7)	13 (46.4)	23	0.50
Medical school year				
MS1	18 (64.2)	24 (85.7)	42	1.0
MS2	10 (35.7)	2 (7.1)	12	0.02*
MS3	0 (0.0)	2 (7.1)	2	1.0
Ethnicity				
White	13 (46.4)	13 (46.4)	26	1.0
Asian	11 (39.3)	11 (39.3)	22	1.0
Hispanic	2 (7.1)	1 (3.6)	3	1.0
Black	1 (3.6)	4 (14.3)	5	1.0

Using a Fisher’s exact test, there was no inferiority seen between the Traditional and Tele-ultrasound groups. Each question from the questionnaire was evaluated as a separate Fisher’s exact test, and the results can be seen in Table 2. In 12 of the 15 parameters, there was no differences in performance between the Traditional and Tele-ultrasound groups. In the remaining three questions, there were slight differences in performance, but none were considered significant with a *p* value of 0.491 (significant < 0.05).

**Table 2:** Number of participants in both Traditional and Remote groups able to complete specified exam elements; Fisher’s exact test statistic performed on each question to determine whether significant difference exists between two study groups

Variable	Completed successfully		Fisher’s exact test statistic
	Traditional (n = 28)	Remote (n = 28)	
FAST exam			
Q1—probe selection	26 (92.85)	28 (100)	0.491
Q2—Morison’s pouch	28 (100)	28 (100)	1.000
Q3—R kidney, liver, diaphragm	28 (100)	28 (100)	1.000
Q4—urinary bladder, peritoneal cavity	28 (100)	28 (100)	1.000
Q5—spleen, L kidney, diaphragm	28 (100)	26 (92.85)	0.491
Q6—4-chamber view of heart	28 (100)	28 (100)	1.000
DVT exam			
Q1—probe selection	28 (100)	27 (96.4)	1.000
Q2—common femoral a/v	28 (100)	27 (96.4)	1.000
Q3—femoral a/v	27 (96.4)	28 (100)	1.000
Q4—popliteal a/v	27 (96.4)	28 (100)	1.000
Q5—performed compression	28 (100)	28 (100)	1.000
Vascular access			
Q1—probe selection	28 (100)	28 (100)	1.000
Q2—short-axis cannulation	26 (96.4)	28 (100)	0.491
Q3—long-axis cannulation	27 (96.4)	28 (100)	1.000

**Fig. 1:**
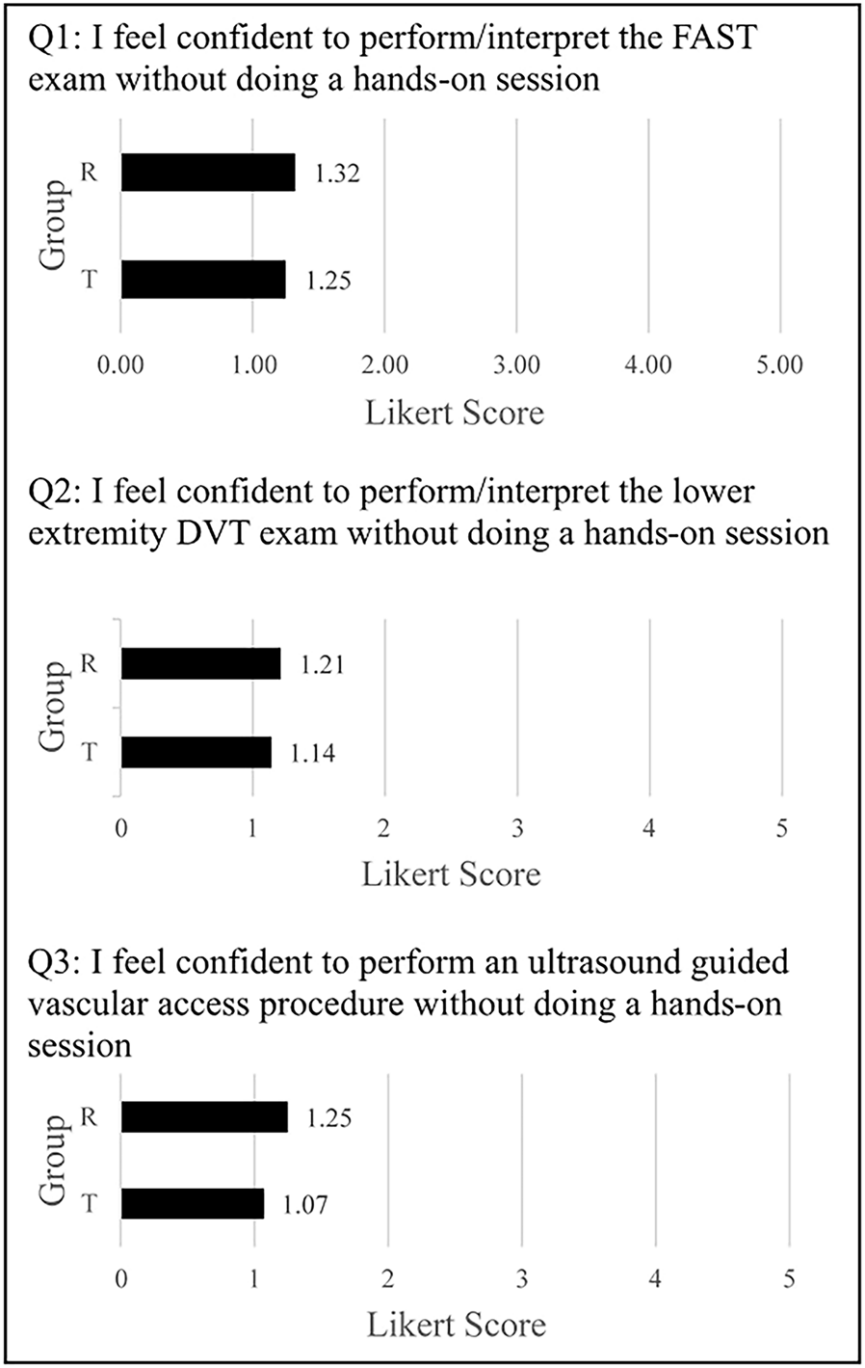
Pre-Assessment Questionnaire answers for the Traditional (T) and Tele-ultrasound (R) groups. Participants rated their confidence with the Likert scale of 1 (strongly agree) to 5 (strongly disagree). Averages between the two groups are shown here. No significant differences were found (*p* < 0.05)

Pre-assessment surveys utilized the Likert scale in order to determine the level of ability that participants had in ultrasound before the study. These questions were used to exclude participants with significant (defined as over 20 lifetime hours of experience) ultrasound experience. Additionally, if participants had any experience in the studied skills, as signified by a “yes” answer on the pre-assessment survey, they were also excluded from the study. Three additional questions asked participants to rate their confidence in ability to perform the three tested skills, utilizing a Likert scale. It was found that there was no significant difference in scores between the Traditional and Tele-ultrasound groups, as seen in Fig. 1, as calculated using a Mann–Whitney U test (*p* < 0.05).

**Fig. 2:**
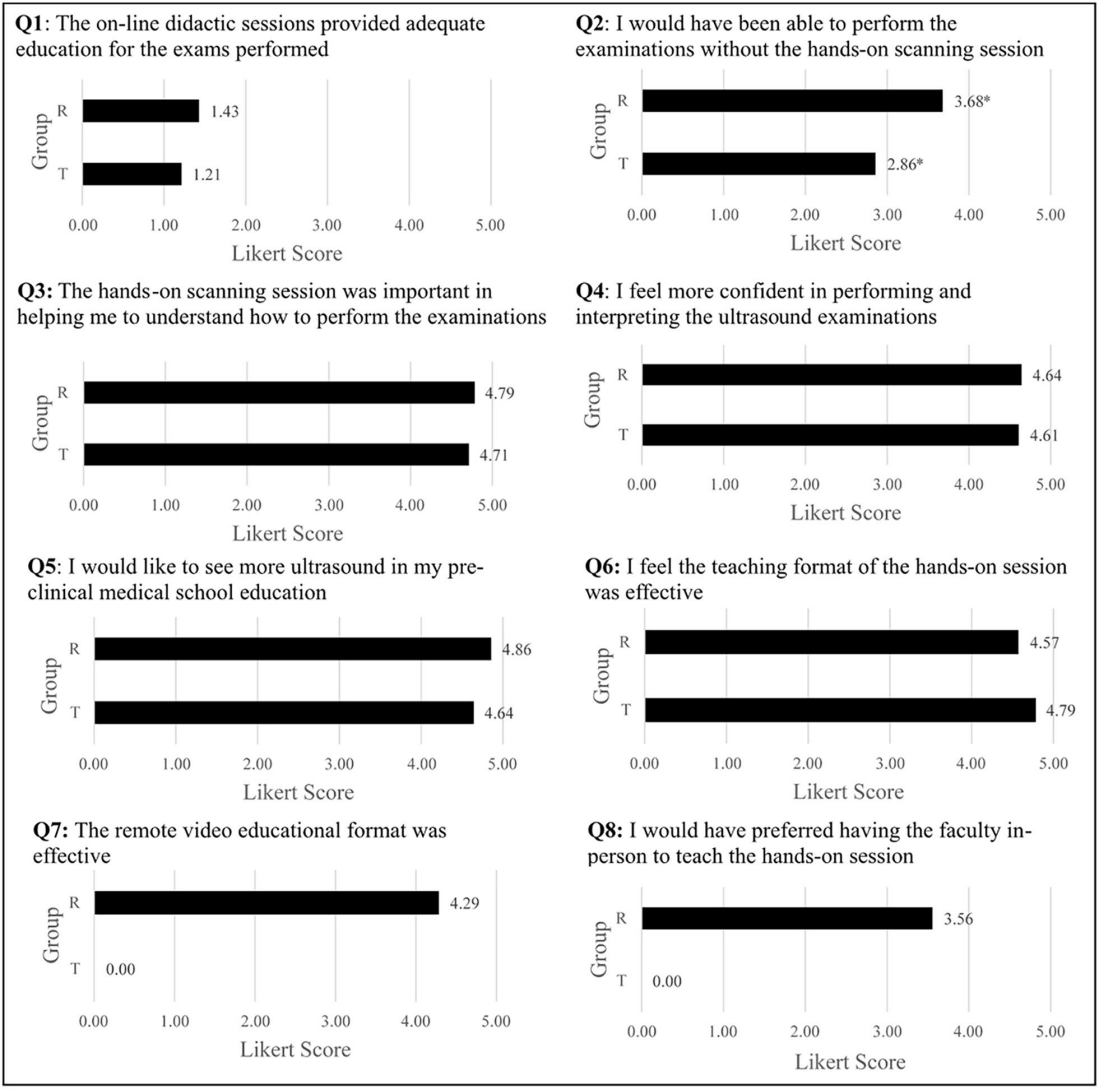
Post-Assessment Questionnaire answers for the Traditional (T) and Tele-ultrasound (R) groups. Participants of both groups rated their confidence in their abilities after their training session, as well as opinions on the pre-session education materials

Post-assessment surveys (seen in Additional file 1: Appendix S3 and S4) were utilized to assess changes in participant confidence in the skills post-teaching and assessment, once again utilizing a Likert Scale. These were also analyzed using a Mann–Whitney U test. Only question 1, referring to whether the online educational modules adequately provided background for the exams tested, showed a significant difference (*p* < 0.05, *p* < 0.001). In addition, the Tele-ultrasound group had two additional questions on the effectiveness of remote video education (average score 4.29/5), and whether the students of this group would have preferred in-person teaching (average score 3.56/5). The full results of this assessment are seen in Fig. 2.

## Discussion

As an imaging modality, POCUS has wide application across a multitude of fields including internal medicine, emergency medicine, obstetrics, critical care, sports medicine, surgery, and rheumatology [7]. Despite widespread interest in learning ultrasound among U.S. medical students, challenges remain in its implementation into curriculums with the number of trained faculty as a limiting factor [5, 14]. Low-cost tele-ultrasound setups such as multiple-fixed cameras, audio in conjunction with a live ultrasound stream, and smartphones have been explored as means of ultrasound training [4]. Poland et al. 2018 demonstrated that tele-ultrasound has a place in medical student education as an effective means of learning new sonography skills [15, 16]. Therein lies an opportunity to build upon these previous studies.

Our results demonstrated that tele-ultrasound instruction was non-inferior with respect to in-person teaching and that there was no statistically significant difference between the two (per the Fisher’s exact test statistic) in the education of ultrasound-naive medical students. The study design focused on what we consider to be beginner ultrasound education fundamentals: being able to recognize anatomical structures, the general applications of each probe, and hand technique. More technical device operation (such as adjusting gain, switching probes in the software, etc.) was not included in the experiment given the nuances as well as variability between manufacturers devices.

From Q8 of the post-assessment questionnaire (“I would have preferred having the faculty in-person to teach the hands-on session”, Additional file 1: Appendix S3), the learners in the Tele-ultrasound group slightly leaned towards preferring to have had the lessons taught in person with an average score of 3.56 (3 being defined as neutral). Nevertheless, Q7 (“The remote video educational format was effective”, see Additional file 1: Appendix S3) indicated that they believed remote learning was an effective means (avg. 4.29) of teaching them the necessary skills for what they were assessed on. We can only speculate as to the relevance of this discrepancy. This may reflect a personal preference from the learners’ perspective, but the significance of the preference for in-person learning is currently unknown given there is no comparison data. Q1 noted a statistically significant difference with regard to the adequacy of the online didactic sessions between the Lumify (3.68) and Traditional (2.86) groups, though reasoning for this discrepancy is unclear.

Remote training has implications for medical education and perhaps even in clinical tele-ultrasound, as it could reduce the need for conventional in-person ultrasound training or expand the reach of instruction to areas lacking in resources [17]. The ability of ultrasound to introduce diagnostic imaging in resource-limited settings in an inexpensive and durable manner has been shown to impact patient care [6, 18, 19]. Moreover, the World Health Organization has recommended ultrasound for use in developing nations whose ultrasound operators have received 3–6 months of training and participated in 300–500 examinations as part of their radiology initiative, further emphasizing the need for increased access to training by qualified sonographers and physicians [20]. Considering the ongoing COVID-19 pandemic, this research also raises the possibility of the use of tele-ultrasound in education and clinical environments where limiting provider exposure may be key. With medical schools across the country adapting to a virtual learning format, this technology could have use in ensuring students are still getting hands-on exposure to ultrasonography, while limiting in-person contact. Future directions include examining the efficacy of using tele-ultrasound in teaching new techniques to practicing physicians as well as in honing pre-existing skills. From an instructional standpoint, the three ultrasound examinations that were taught in this study are basic techniques with a narrow scope. However, it is worth further investigating if tele-ultrasound can be used to teach more technically challenging ultrasound examinations such as imaging of the hepato-biliary tract, assessing lung function, obtaining the four cardiac windows, and whether it can be used to teach more complex ultrasound-guided procedures. Moreover, it would be worth exploring the efficacy and feasibility of tele-ultrasound in the use of education of more advanced techniques such as caliper placement for a thickened gall bladder wall, accessing M-mode, and more complicated ultrasound-guided procedures. This could be further explored using participants that have more advanced ultrasound training prior to study initiation.

### Limitations

This study had several distinct limitations regarding both the participant pool and the non-inferiority study design. Participants were all given access to the same modules, but there was no way to ensure that they were completed in entirety before their training sessions or how much knowledge was retained. It is worth noting that the composition of medical students varied in several ways and may have served as a confounding variable. Many participants were first years, only several months into their education, so the standards of proficiency used in this study were more lenient and could explain the performance of both groups. For example, proficiency credit was given for the ability to recognize an anatomical relationship, without significant emphasis on optimal image quality given the latter would represent criteria for grading of more advanced users. The groups were similar with respect to their degree of ultrasound experience as denoted in Fig. 1. However, the distribution of preclinical education levels was unequal, with the Traditional group having far more second and third-year students (35% of the group) than the Tele-ultrasound group (14%). This may be a confounding variable given second-year or third-year medical students likely have an increased understanding of general anatomy and anatomical relationships, even if they had not been exposed to anatomy via ultrasound. Students were also pooled from three different medical schools in the Northeast Ohio area and differences in the respective anatomy curriculums may have played a role.

While we attempted to keep our student investigator consistent with respect to their roles as standardized patients and administrators of the post-teaching session skill demonstration to account for anatomical variation and to minimize any grading bias, the individual schedules and time constraints of the student investigators occasionally varied between sessions. From a study design standpoint, another limitation of the study was the minimal education about operating the individual ultrasound machines. We opted to overlook this aspect due to our study design philosophy focusing on general proficiency as opposed to device proficiency and logistic availability/access of machines. Given the logistics of acquiring ultrasound machines, different machines were used during different sessions, and it was also deemed unfeasible for ultrasound images to be recorded from every participant to be assessed by a blind grader.

## Conclusions

POCUS has become increasingly integrated into medical education given the growing role of evaluative and procedural techniques in practice today. Tele-ultrasound is a feasible way to teach and expand upon POCUS skills including both diagnostic and procedural techniques. This study proved a non-inferiority in the use of tele-ultrasound technology in teaching ultrasound in beginners, and provides a foundation to look further into the use of technology in the education of medical professionals.

## Supplementary Information

**Additional file 1.** Appendices including: pre-training assessment, exam criteria and data selection, and post-training assessments for both traditional and remote study groups

## Data Availability

Data supporting the findings of this paper can be found by contacting the corresponding author.
