# Supplementary material for: Innovations with tele-ultrasound in education sonography: the use of tele-ultrasound to train novice scanners
Source: Ultrasound J. 2021 Feb 14;13:6. doi: 10.1186/s13089-021-00210-0 (PMC7882469; doi:10.1186/s13089-021-00210-0)
Supplement: Supplementary file 1 — Additional file 1. Appendices including: pre-training assessment, exam criteria and data selection, and post-training assessments for both traditional and remote study groups [file 13089_2021_210_MOESM1_ESM.docx]

**APPENDICES**

**Appendix 1: Pre-training Assessment**

**HANDS ON SESSION PRE ASSESSMENT**

I have had previous training in ultrasound Yes No

(If yes, how many hours of training?) _____________________

I have performed ultrasound examinations previously Yes No

(If yes, how many exams have you performed?) _____________

(If yes, have you performed an ultrasound guided procedure?) Yes No

(If yes, have you performed a FAST Exam?) Yes No

(If yes, have you performed a lower extremity venous compression exam?) Yes No

I feel confident to perform/interpret the FAST exam without doing a hands-on session

1 2 3 4 5

I feel confident to perform/interpret the lower extremity DVT exam without doing a hands-on session

1 2 3 4 5

I feel confident to perform an ultrasound guided vascular access procedure without doing a hands-on session

1 2 3 4 5

**Appendix 2: Exam Criteria and Data Selection**

**REMOTE ULTRASOUND RESEARCH PROJECT**

**DATA**

**Study Participant number:**

DVT:

1. Correct transducer selection Yes No
2. Identifies common femoral artery/vein Yes No
3. Identifies femoral artery/vein Yes No
4. Identifies popliteal artery/vein Yes No
5. Performs venous compression of common femoral vein, femoral vein, and popliteal vein Yes No

FAST Exam:

1. Correct transducer selection Yes No
2. Identifies Morison’s Pouch in perihepatic window Yes No
3. Identifies right Kidney, liver, and diaphragm in perihepatic window Yes No
4. Identifies urinary bladder and peritoneal cavity in pelvic window Yes No
5. Identifies spleen, left kidney, and diaphragm in perisplenic window Yes No
6. Identifies 4 chamber view of heart in pericardial window Yes No

Procedural Guidance

1. Correct transducer selection Yes No
2. Performs vessel cannulation of phantom (short-axis, out-of-plane) Yes No
3. Performs vessel cannulation on phantom (in-plane, long axis) Yes No

**Appendix 3: Post-Training Tele-ultrasound Assessment**

**POST-TRAINING ASSESSMENT**

**LUMIFY UNITS**

Q1 The on-line didactic sessions provided adequate education for the exams performed

1 2 3 4 5

Q2 I would have been able to perform the examinations without the hands-on scanning session

1 2 3 4 5

Q3 The hands-on scanning session was important in helping me to understand how to perform the examinations

1 2 3 4 5

Q4 I feel more confident in performing and interpreting the ultrasound examinations

1 2 3 4 5

Q5 I would like to see more ultrasound in my pre-clinical medical school education

1 2 3 4 5

Q6 I feel the teaching format of the hands-on session was effective

1 2 3 4 5

Q7 The remote video educational format was effective

1 2 3 4 5

Q8 I would have preferred having the faculty in-person to teach the hands-on session

1 2 3 4 5

**Appendix 4: Post-Training In-Person Assessment**

**POST-TRAINING ASSESSMENT**

**IN-PERSON**

Q1 The on-line didactic sessions provided adequate education for the exams performed

1 2 3 4 5

Q2 I would have been able to perform the examinations without the hands-on scanning session

1 2 3 4 5

Q3 The hands-on scanning session was important in helping me to understand how to perform the examinations

1 2 3 4 5

Q4 I feel more confident in performing and interpreting the ultrasound examinations

1 2 3 4 5

Q5 I would like to see more ultrasound in my pre-clinical medical school education

1 2 3 4 5

Q6 I feel the teaching format of the hands-on session was effective

1 2 3 4 5
